# Spontaneous Esophageal Rupture or Boerhaave’s Syndrome

**DOI:** 10.5334/jbsr.1882

**Published:** 2020-01-15

**Authors:** François Carrozza, Cristina Dragean

**Affiliations:** 1 UCL, Saint-Luc, BE

**Keywords:** spontaneous esophageal rupture, Boerhaave’s syndrome, computed tomography

## Abstract

**Teaching point:** Boerhaave syndrome is a very rare life-threatening surgical emergency, often misdiagnosed at the patient’s admittance.

## Case

**Figure 1:**
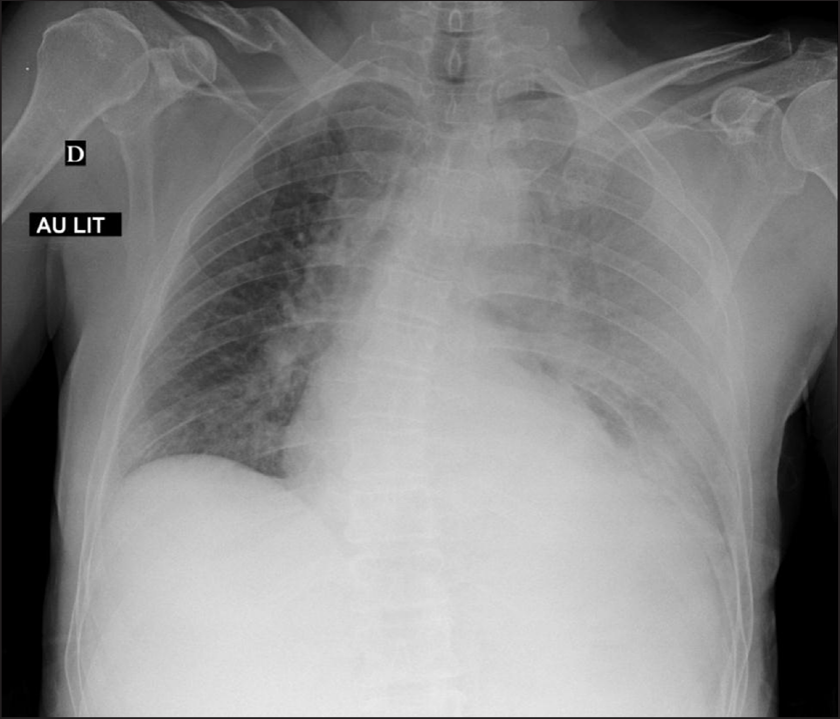


**Figure 2:**
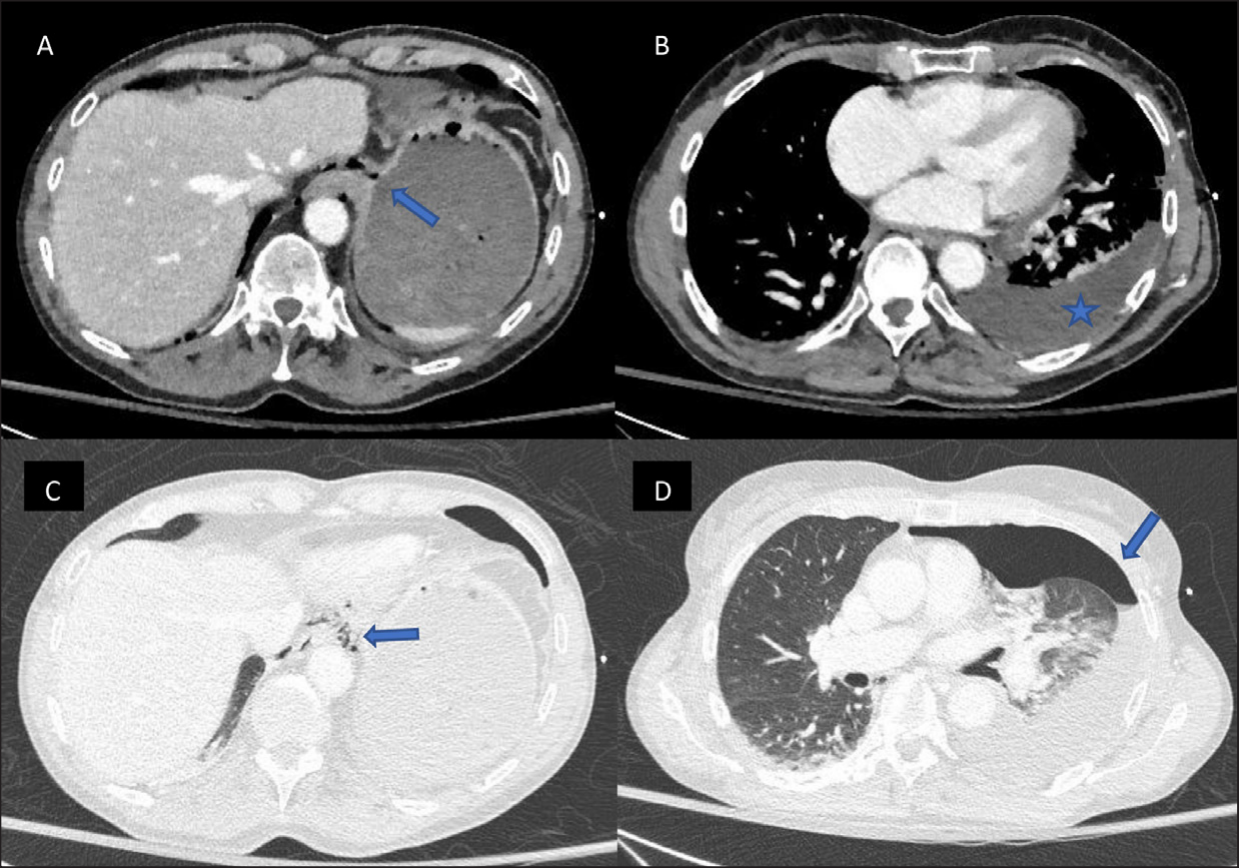


**Figure 3:**
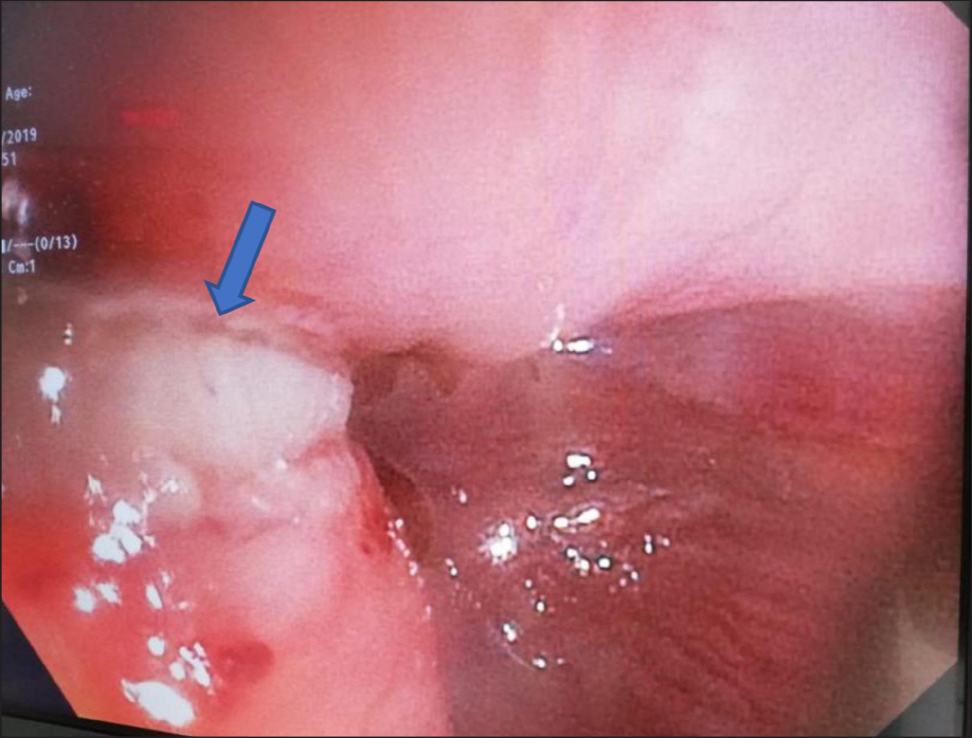
(Courtesy of Pr T. Moreels).

A 65-year-old woman was admitted to the emergency service, presenting with diffuse abdominal pain and back pain. The patient had a recent history of nausea, and she experienced a severe pain after an episode of vomiting. On physical examination, a diffuse abdominal sensibility was reported, mostly in the right upper quadrant. There was no fever and cardiopulmonary auscultation was normal. The blood pressure, respiratory frequency and oxygen saturation were 120/64 mmHg, 15/min and 100%, respectively. A few times later, respiratory frequency and oxygen saturation were 44/min and 94% respectively, with normal blood pressure. Chest radiography was performed, showing a veil-like increased density of the left hemithorax, consistent with pleural effusion (Figure 1). Contrast-enhanced computed tomography (CT) was then performed to exclude an aortic dissection, showing a pneumomediastinum (Figure 2C) with left hydropneumothorax (Figure 2B–2D). A focal perforation was suspected on antero-lateral side of the gastroesophageal junction (Figure 2A). The diagnosis of spontaneous esophageal rupture (or Boerhaave’s syndrome) was suggested. A preoperative gastroscopy assessed the diagnosis, showing a 3 cm-length perforation at the gastro-esophageal junction (greater curvature’s side of the stomach, beginning at 9 o’clock superficially and extending posteriorly to the esophagus – Figure 3). The patient was subsequently operated on (primary repair of the esophagus).

## Comment

Boerhaave syndrome or spontaneous esophageal perforation is very rare (estimated incidence 1/6000) surgical emergency, life-threatening, with high morbidity and mortality. It is most often diagnosed in men aged 50–70 years, after heavy meal ingestion combined with abundant alcohol consumption. It occurs when there is an incomplete relaxation of the cricopharyngeal muscle during vomiting, resulting in abruptly increased intraluminal pressure enough to rupture the esophagus. The perforation is mostly located on the left posterior aspect of the distal esophagus, 2–3 cm proximally to the gastro-esophageal junction [1]. Many patients present with atypical symptoms and findings on physical exam are often non-specific. The “Macklers triad” with vomiting, lower chest pain and subcutaneous emphysema is present only in a minority of patients. Therefore, it is often, as in our case, misdiagnosed as acute aortic syndrome, pericarditis, myocardial infarction, pulmonary embolus, spontaneous pneumothorax or perforated peptic ulcer. Untreated cases may rapidly progress to infectious mediastinitis and septic shock within 24–48 h.

Chest radiography is often abnormal (but may be normal), with non-specific findings such as left pleural effusion and left pneumothorax, pneumomediastinum and gas in the soft tissue spaces of the chest wall and the neck. CT may show indirect signs of Boerhaave’s, such as esophageal wall edema and peri-esophageal collections. Esophagus tear can be seen or may require CT-esophagography.
